# *Listeria Monocytogenes* La111 and *Klebsiella Pneumoniae* KCTC 2242: Shine-Dalgarno Sequences

**Published:** 2014

**Authors:** Gholamreza Motalleb

**Affiliations:** Department of Biology, University of Zabol, Zabol, Iran.

**Keywords:** Molecular biology, genomics, microbiology, Shine-Dalgarno sequences

## Abstract

Listeria monocytogenes can cause serious infection and recently, relapse of listeriosis has been reported in leukemia and colorectal cancer, and the patients with *Klebsiella pneumoniae* are at increased risk of colorectal cancer. Translation initiation codon recognition is basically mediated by Shine-Dalgarno (SD) and the anti-SD sequences at the small ribosomal RNA (ssu rRNA). In this research, Shine-Dalgarno sequences prediction in Listeria monocytogenes La111 and *Klebsiella pneumoniae* KCTC 2242 was investigated. The whole genomic sequence of Listeria monocytogenes La111 and *Klebsiella pneumoniae* KCTC 2242 were retrieved from http://www.ncbi.nlm.nih.gov/ (Listeria monocytogenes La111 NCBI Reference sequence: NC_020557; *Klebsiella pneumoniae* KCTC 2242 NCBI Reference sequence: CP002910) in order to be analyzed with DAMBE software and BLAST. The results showed that the consensus sequence for *Klebsiella pneumoniae* KCTC 2242 was CCCCCCCUCCCCCUCCCCCUCCUCCUCCUUUUUAAAAAAGGGGAAAAACC and for *Listeria monocytogenes* La111 was CCCCCCCUCCCCCUUUCCCUCCUAUUCUUAUAAAAGGGGG-GGGGUUCAC. The P_SD_ was higher in *Listeria monocytogenes* La111 compared to *Klebsiella pneumoniae *KCTC 2242 (0.9090> 0.8618). The results showed that Nm in Listeria monocytogenes La111 was higher than *Klebsiella pneumoniae* KCTC 2242 (4.5846> 4.4862). Accurate characterization of SD sequences may increase our knowledge on how an organism’s transcriptome is related to its cellular proteome.

Possible correlation and associations of rare bacteria with serious disease, especially cancer and laboratory isolations of these organisms in these patients have initiated the studies of pathogenetic significance of the agent (1).* Listeria monocytogenes* is an aerobic, gram-positive bacillus that has become an important pathogen in the 21st century (2). Transplantation patients, persons with neoplastic disease, immunocompromised subjects, pregnant women (2), and HIV patients (3) are at high risk. To our knowledge, infection relapse of *Listeria monocytogenes* is rare, but relapse of listeriosis has been reported in leukemia and colorectal cancer (2, 4). *Klebsiella pneumoniae* is a gram-negative, anaerobic, and rod shaped bacterium. Neoplastic diseases are common in patients with nosocomial *Klebsiella pneumoniae *bacteraemia (5). Henao-Martínez et al. reported that *E. coli* and *Klebsiella pneumoniae* are especially prevalent in patients with gastrointestinal (GI) and lung cancers (6). Due to their abilities to cause basic cellular functional changes and attack host defense mechanisms, these bacteria have become a model for host pathogen interactions (7). 

A molecular machine like ribosome translates the genetic code from messenger RNA into an amino acid sequence by RNA selection, peptide bond formation and translocation (8). Protein synthesis by ribosomes takes place on a linear substrate but at variable speeds. Transient pausing of ribosomes can impact a variety of co-translational processes, including protein targeting and folding. These pauses are influenced by the sequence of the mRNA. Thus, redundancy in the genetic code allows the same protein to be transla-ted at different rates (9). mRNA sequences contain many AUG. How does the translation machinery distinguish which one is the initiation codon? Initial positioning of the ribosome on mRNA involves the recognition of a purine rich sequence, known as the Shine Dalgarno (SD) sequence, located upstream of the AUG initiation codon on the mRNA (8). 

In 1974, Shine and Dalgarno sequenced the 3' end of *Escherichia coli’s* 16S ribosomal RNA (rRNA) and observed that part of the sequence, 5'–ACCUCC–3', was complementary to a motif, 5'–GGAGGU–3', located 5' of the initiation codons in several messenger RNAs (mRNAs) (9). They combined this observation with previously published experimental evidences and suggested that complementarity between the 3' tail of the 16S rRNA and the region 5' of the start codon on the mRNA was sufﬁcient to create a stable, double-stranded structure that could position the ribosome correctly on the mRNA during translation initiation. The motif on the mRNAs, 5'– GGAGGU–3', and variations on it that are also complementary to parts of the 3' 16S rRNA tail, have since been referred to as the Shine–Dalgarno (SD) sequence. Shine and Dalgarno’s theory was bolstered by Steitz and Jakes in 1975 (10) and eventually experimentally veriﬁed in 1987, by Hui and de Boer (11) and Jacob et al. (12).The SD sequence has been established by experimental evidence that came from mutation studies. Unfortunately, experiments are tedious and only a few mutated SD sequences have been examined. Biopharmaceutical studies are highly interested in improving translation efficiency (13). In the present study, we tried to find the best possible SD for translation in *Listeria mono-cytogenes* La111, and *Klebsiella pneumoniae* KCTC 2242 through DAMBE software and BLAST analyzes.

## Materials and methods

This research started in Spring 2013 and data analyses were performed at bioinformatics facility of Faculty of Sciences in Zabol University, Iran. *Listeria monocytogenes* La111 (NCBI Reference sequence: NC_020557) and *Klebsiella pneumoniae* KCTC 2242 (NCBI Reference sequence: CP002910) genome sequences were retrieved from http://www.ncbi.nlm.nih.gov. Fifty nucleotides upstream of the initiation coding sequences from each gene were extracted and position weight matrix (PWM) was employed to determine the SD sequence and location by the FASTA algorithm using DAMBE (14, 15). PWM is computed as:

${\mathrm{PWM}}_{\mathrm{ij}}=\log_{2}\left(\frac{p_{\mathrm{ij}}}{p_{i}}\right)$ (1) where i= 1, 2, 3 and 4 refer to A, C, G and U, respectively, and j is the site index, and *p*_i _ is the background frequency of nucleotide *i*, and *p*_ij_ is the site specific nucleotide frequency for nucleotide *i *at site *j*.

## Results

**Fig 1:**
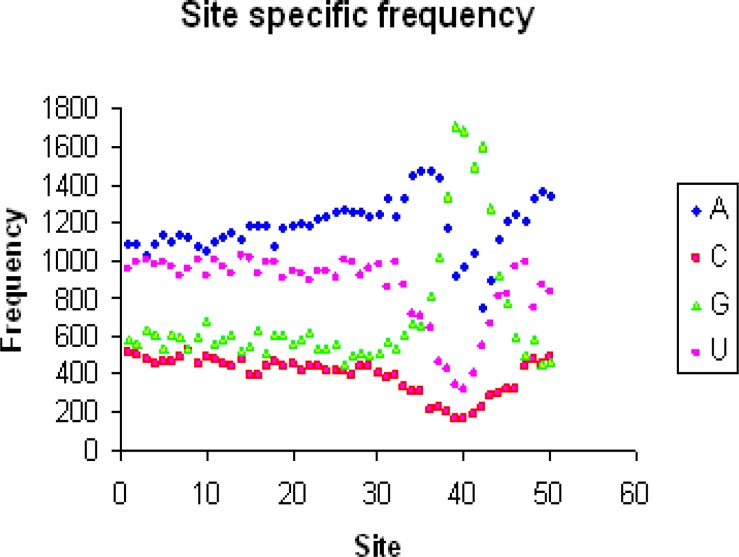
Site specific frequency scatter diagram of *Klebsiella pneumoniae* KCTC 2242

**Fig 2:**
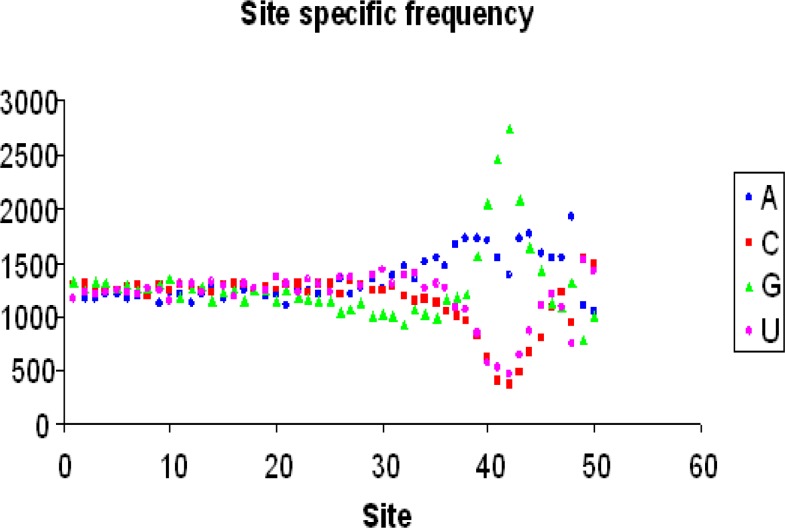
Site specific frequency scatter diagram of *Listeria monocytogenes* La111

The position and sequence of Shine-Dalgarno as a functional motif was investigated in *Listeria monocytogenes* La111 and *Klebsiella pneumoniae* KCTC 2242 in order to find genetic motifs by DAMBE. SD sequence is often characterized by altered nucleotide frequencies (15). Table 1 and Figure 1 show the site specific frequency for *Klebsiella pneumoniae* KCTC 2242. Also Table 2 and Figure 2 show the site specific frequency for *Listeria monocytogenes* La111. PWM analysis showed that the consensus sequence for *Klebsiella pneumoniae* KCTC 2242 was CCCCCCCUCCCC-CUCCCCCUCCUCCUCCUUUUUAAAAAAG-GGGAAAAACC (Table 3) and for *Listeria mono-cytogenes* La111 was CCCCCCCUCCCCCUUU-CCCUCCUAUUCUUAUAAAAGGGGGGGGG-UUCAC (Table 4). FASTA algo-rithm analysis search output for *Klebsiella pneumoniae* KCTC 2242 and *Listeria monocytogenes* La111 has been shown in Tables 5 and 6. The results showed that the PSD was higher in *Listeria monocytogenes* La111 compared to *Klebsiella pneumoniae* KCTC 2242 (0.9090> 0.8618) (Table. 7). In *Listeria monocytogenes* La111, 2600 genes and in *Klebsiella pneumoniae* KCTC 2242, 3830 genes have Nm (the number of matched sites) ≥3 and Sm (the start of the match) within the range of 30 and 45 (NSD) and the proportion of 50 mers with the SD sequences is PSD = NSD/N (Table 7). In Tables 5 and 6, the second column being Nm or number of matched sites between the SD sequences and the 50 mers and third column is Sm or start of the match. 

**Table 1:** Site specific frequencies analysis of *Klebsiella pneumoniae* KCTC 2242

**Site**	**A**	**C**	**G**	**U**
1	1153	1289	1333	1148
2	1143	1313	1253	1214
3	1152	1247	1334	1190
4	1187	1222	1311	1203
5	1199	1247	1243	1234
6	1151	1269	1289	1214
7	1182	1299	1251	1191
8	1245	1174	1256	1248
9	1106	1301	1290	1226
10	1212	1237	1345	1129
11	1187	1286	1163	1287
12	1108	1274	1250	1291
13	1196	1243	1280	1204
14	1275	1204	1128	1316
15	1161	1295	1203	1264
16	1168	1321	1260	1174
17	1225	1290	1125	1283
18	1213	1235	1226	1249
19	1177	1281	1230	1235
20	1194	1248	1128	1353
21	1097	1299	1229	1298
22	1231	1308	1168	1216
23	1240	1216	1145	1322
24	1184	1314	1134	1291
25	1271	1303	1128	1221
26	1324	1201	1039	1359
27	1185	1335	1060	1343
28	1247	1291	1115	1270
29	1325	1232	992	1374
30	1258	1230	1011	1424
31	1368	1264	992	1299
32	1473	1169	911	1370
33	1339	1138	1052	1394
34	1507	1158	1007	1251
35	1541	1121	965	1296
36	1478	1035	1157	1253
37	1674	997	1178	1074
38	1734	962	1183	1044
39	1723	807	1562	831
40	1702	611	2053	557
41	1552	397	2456	518
42	1371	352	2738	462
43	1738	472	2084	629
44	1775	659	1640	849
45	1594	804	1430	1095
46	1541	1069	1122	1191
47	1556	1205	1082	1080
48	1929	942	1315	737
49	1088	1544	765	1526
50	1040	1484	993	1406

* Site-specific counts with a window of 50 bases. A: adenine; C: cytosine; G: guanine; U: uracil. For example at site 1, A, 1153 times, C, 1289 times has been replicated and so on. The site specific frequencies can be used to derive a PWM to rapidly scan other sequences.

**Table 2:** Site specific frequencies analysis of *Listeria monocytogenes* La111.

**Site**	**A**	**C**	**G**	**U**
1	1082	509	586	954
2	1079	495	563	994
3	1020	482	629	1000
4	1090	453	609	979
5	1136	469	536	990
6	1092	466	612	961
7	1135	491	592	913
8	1123	520	538	950
9	1075	455	600	1001
10	1051	492	675	913
11	1099	472	559	1001
12	1125	452	587	967
13	1150	447	604	930
14	1105	475	525	1026
15	1177	390	552	1012
16	1182	391	633	925
17	1183	441	512	995
18	1077	466	604	984
19	1172	447	605	907
20	1175	452	565	939
21	1190	422	589	930
22	1181	445	615	890
23	1210	442	537	942
24	1231	418	538	944
25	1253	414	555	909
26	1263	415	457	996
27	1246	391	503	991
28	1252	442	518	919
29	1232	437	504	958
30	1244	402	513	972
31	1321	380	572	858
32	1223	391	531	986
33	1320	335	603	873
34	1446	308	667	710
35	1461	313	659	698
36	1467	212	813	639
37	1430	228	1013	460
38	1171	199	1330	431
39	918	164	1708	341
40	961	172	1680	318
41	1039	194	1492	406
42	752	224	1602	553
43	898	283	1279	671
44	1108	302	913	808
45	1204	325	778	824
46	1241	325	598	967
47	1204	443	498	986
48	1321	482	581	747
49	1354	448	453	876
50	1341	487	463	840

* Site-specific counts with a window of 50 bases. A: adenine; C: cytosine; G: guanine; U: uracil. For example at site 1, A, 1082 times, C, 509 times has been replicated and so on. The site specific frequencies can be used to derive a PWM to rapidly scan other sequences.

## Discussion

This study was conducted in order to find SD sequences in bacterial species by focusing on *Klebsiella pneumoniae* KCTC 2242 (representing gram negative bacteria) and *Listeria monocytogenes* La111 (representing gram positive bacteria) by performing a simulation study. Fifty nucleotides upstream of the CDS or coding sequences were extracted from each gene, then position weight matrix or PWM was used in order to find the SD sequence location (15). We studied and focused on those results or signals limited to the 20 nucleotides upstream of the CDSs. After that, the 16S rRNA or small subunit rRNA was extracted from the genome and we used the last 8 nucleotides in order to find the best match to the upstream sequence by the FASTA algorithm to rank SDs by the number of matched sites (matching strength or simply MS). Translation initiation is the limiting step and a main phase in gene expression in bacteria (16, 17). 

As messenger RNAs has many AUG sequences, the main question is: How does the translational machinery knows which one is the initiation codon? In eukaryotes, this is accomplished by the scanning of the small ribosomal subunit which finds the first AUG and in prokaryotes, mainly through the matching between the Shine Dalgarno (SD) sequences located about 9 nucleotides upstream of the translation initiation codon and the anti-SD sequences at the 3’ end of ssu rRNA or small ribosomal RNA (18). The SD sequence may be defined by experimental tests showing the SD sequences related to the most accurate positioning of the ribosome at the translation initiation site and the best documents correspond to mutagenesis studies. On the other hand, first, many genes in bacteria (gram negative) have not any short or even trace of a SD sequence and second, when SD sequences are present, their location is very often variable. The ribosomal protein S1 in gram negative bacteria helps to locate TIC or translation initiation codon by binding to AU-rich sequences located 15-30 nucleotides upstream of start codon (18). We called it as S1 hypothesis. For efficient translation initiation, Nm should be four or more and SD sequence may be defined as one with Nm ≥3 and 31 ≤Sm ≤45 (11, 14). For mRNAs that have a weak or no SD sequence, the S1 protein is necessary to recognize the initiation codon and therefore, reduces the importance of a strong SD sequence and may allow the SD sequence to degrade. In gram-positive bacteria, either they do not have the S1 protein, or have an “S1 protein” that is not conserved and probably is not used to recognize the initiation codon. This important fact suggests that in gram positive bacteria TIC localization may be more dependent on the SD sequence than in the gram negative bacteria (18). Therefore if an essential protein-coding gene in *Listeria monocytogenes* La111 had lost the SD sequence, so it may not be properly translated and the mutant will be selected against and in *Klebsiella pneumoniae* KCTC, genes may be more tolerant to mutations obliterating the SD sequence (18). These important facts caused to lead us to test two predictions: (1) the presence of a greater proportion of SD-containing protein-coding genes in *Listeria monocytogenes* La111 than in *Klebsiella pneumoniae *KCTC, and (2) the existence of better matches between the SD sequence in mRNA and the anti-SD sequence in *Listeria monocytogenes* La111 than in *Klebsiella pneumoniae* KCTC. The results showed that the P_SD_ is greater in *Listeria monocytogenes* La111 than in *Klebsiella pneumoniae* KCTC 2242 and this agreed with one of our predictions that *Listeria mono-cytogenes* La111 genes should more likely have the SD sequence than those in *Klebsiella pneumoniae* KCTC 2242. The second of our prediction was that Nm should be higher for *Listeria monocytogenes* La111 genes than *Klebsiella pneumoniae* KCTC 2242 genes. Our results confirmed this hypothesis (4.5846>4.4862). Thus, it can be concluded that accurate characterization of SD sequences may increase our knowledge on how an organism’s transcriptome is related to its cellular proteome.

**Table 3:** PWM analysis of *K. pneumoniae* KCTC 2242. The consensus sequence is: CCCCCCCUC-CCCCUCCCCCUCCUCCUCCUUUUUAAAAAAGGGGAAAAACC

**Site**	**A**	**C**	**G**	**U**
1	0.2105-	0.1726	0.0677	0.0304-
2	0.2230-	0.1992	0.0215-	0.0502
3	0.2117-	0.1248	0.0688	0.0214
4	0.1685-	0.0956	0.0437	0.0371
5	0.1540-	0.1248	0.0331-	0.0738
6	0.2130-	0.1500	0.0193	0.0502
7	0.1746-	0.1837	0.0238-	0.0227
8	0.0997-	0.0378	0.0181-	0.0901
9	0.2705-	0.1859	0.0204	0.0644
10	0.1385-	0.1132	0.0807	0.0545-
11	0.1685-	0.1692	0.1290-	0.1345
12	0.2679-	0.1557	0.0250-	0.1389
13	0.1576-	0.1201	0.0092	0.0383
14	0.0654-	0.0742	0.1731-	0.1666
15	0.2005-	0.1792	0.0803-	0.1085
16	0.1918-	0.2079	0.0135-	0.0019
17	0.1231-	0.1737	0.1770-	0.1300
18	0.1373-	0.1108	0.0529-	0.0912
19	0.1807-	0.1636	0.0483-	0.0750
20	0.1601-	0.1259	0.1731-	0.2066
21	0.2823-	0.1837	0.0494-	0.1467
22	0.1160-	0.1937	0.1229-	0.0526
23	0.1055-	0.0885	0.1515-	0.1732
24	0.1722-	0.2003	0.1655-	0.1389
25	0.0699-	0.1881	0.1731-	0.0585
26	0.0110-	0.0706	0.2917-	0.2130
27	0.1710-	0.2231	0.2628-	0.1959
28	0.0974-	0.1748	0.1898-	0.1153
29	0.0099-	0.1073	0.3584-	0.2288
30	0.0847-	0.1050	0.3311-	0.2804
31	0.0362	0.1443	0.3584-	0.1479
32	0.1428	0.0316	0.4813-	0.2246
33	0.0053	0.0072-	0.2737-	0.2497
34	0.1757	0.0180	0.3368-	0.0935
35	0.2079	0.0289-	0.3982-	0.1445
36	0.1477	0.1440-	0.1365-	0.0959
37	0.3273	0.1980-	0.1106-	0.1265-
38	0.3781	0.2495-	0.1044-	0.1674-
39	0.3690	0.5029-	0.2964	0.4965-
40	0.3513	0.9042-	0.6907	1.0734-
41	0.2182	1.5259-	0.9493	1.1781-
42	0.0393	1.6994-	1.1060	1.3431-
43	0.3815	1.2764-	0.7123	0.8981-
44	0.4118	0.7951-	0.3667	0.4656-
45	0.2567	0.5083-	0.1691	0.0986-
46	0.2079	0.0974-	0.1808-	0.0227
47	0.2219	0.0754	0.2332-	0.1185-
48	0.5319	0.2798-	0.0481	0.6696-
49	0.2942-	0.4329	0.7332-	0.3802
50	0.3592-	0.3758	0.3570-	0.2620

* PWM sequences scanning with a window of 50 bases, e.g., from site 1 to site 50, from site 2 to site 50, and so on. A: adenine; C: cytosine; G: guanine; U: uracil.

**Table 4:** PWM analysis of *Listeria monocytogenes* La111. The consensus sequence is: CCCCCCCCU-CCCCCUUUCCCUCCUAUUCUUAUAAAAGG-GGGGGGGUUCAC

**Site**	**A**	**C**	**G**	**U**
1	0.1200-	0.3692	0.2661-	0.1573
2	0.1240-	0.3289	0.3239-	0.2165
3	0.2051-	0.2906	0.1640-	0.2252
4	0.1093-	0.2011	0.2106-	0.1946
5	0.0497-	0.2511	0.3947-	0.2107
6	0.1067-	0.2419	0.2035-	0.1678
7	0.0510-	0.3172	0.2514-	0.0939
8	0.0663-	0.4000	0.3894-	0.1512
9	0.1293-	0.2074	0.2321-	0.2267
10	0.1619-	0.3202	0.0622-	0.0939
11	0.0975-	0.2603	0.3342-	0.2267
12	0.0638-	0.1979	0.2637-	0.1768
13	0.0321-	0.1818	0.2225-	0.1205
14	0.0896-	0.2695	0.4246-	0.2622
15	0.0014	0.0149-	0.3523-	0.2424
16	0.0075	0.0112-	0.1549-	0.1128
17	0.0088	0.1623	0.4608-	0.2180
18	0.1266-	0.2419	0.2225-	0.2020
19	0.0047-	0.1818	0.2201-	0.0844
20	0.0010-	0.1979	0.3188-	0.1344
21	0.0173	0.0988	0.2588-	0.1205
22	0.0063	0.1754	0.1965-	0.0571
23	0.0413	0.1656	0.3921-	0.1390
24	0.0661	0.0851	0.3894-	0.1421
25	0.0917	0.0712	0.3445-	0.0876
26	0.1031	0.0747	0.6247-	0.2194
27	0.0836	0.0112-	0.4864-	0.2122
28	0.0905	0.1656	0.4440-	0.1034
29	0.0673	0.1492	0.4835-	0.1633
30	0.0813	0.0288	0.4580-	0.1843
31	0.1679	0.0524-	0.3010-	0.0043
32	0.0567	0.0112-	0.4083-	0.2049
33	0.1668	0.2342-	0.2249-	0.0293
34	0.2983	0.3553-	0.0794-	0.2687-
35	0.3132	0.3321-	0.0968-	0.2933-
36	0.3191	0.8939-	0.2061	0.4207-
37	0.2822	0.7890-	0.5233	0.8946-
38	0.0060-	0.9852-	0.9160	0.9885-
39	0.3570-	1.2641-	1.2768	1.3262-
40	0.2910-	1.1954-	1.2530	1.4268-
41	0.1784-	1.0219-	1.0818	1.0747-
42	0.6446-	0.8145-	1.1844	0.6291-
43	0.3888-	0.4774-	0.8596	0.3502-
44	0.0857-	0.3837-	0.3734	0.0823-
45	0.0341	0.2779-	0.1426	0.0540-
46	0.0778	0.2779-	0.2369-	0.1768
47	0.0341	0.1689	0.5008-	0.2049
48	0.1679	0.2906	0.2785-	0.1955-
49	0.2035	0.1850	0.6374-	0.0343
50	0.1896	0.3054	0.6059-	0.0262-

* PWM sequences scanning with a window of 50 bases, e.g., from site 1 to site 50, from site 2 to site 50, and so on. A: adenine; C: cytosine; G: guanine; U: uracil

**Table 5:** FASTA algorithm representative output of *Klebsiella pneumoniae* KCTC 2242 in some of target sequences.

**Target name**	**Max match**	**Shift**
KPN2242_00005|C803	5	40
KPN2242_00010|960	5	34
KPN2242_00015|2150	5	38
KPN2242_00020|3831	4	36
KPN2242_00025|5238	4	38
KPN2242_00030|C7902	5	39
KPN2242_00035|8097	4	43
KPN2242_00045|C14438	6	5
KPN2242_00050|C15663	5	41
KPN2242_00055|C16560	4	39
KPN2242_00060|C17372	5	19
KPN2242_00065|C17932	6	37
KPN2242_00070|C18339	6	40
KPN2242_00075|C19152	4	39
KPN2242_00080|C20170	5	32
KPN2242_00085|20520	6	40

*First column is some of sequences name; second column is the number of matched sites (Nm) between the SD sequence and the 50mer, and the last column is the start of the match (Sm).

**Table 6:** FASTA algorithm output of *Listeria monocytogenes *La111 in some of target sequences

**Target name**	**Max Match**	**Shift**
BN418_0001|318	5	39
BN418_0002|1867	4	39
BN418_0003|3121	5	34
BN418_0005|4869	4	34
BN418_0006|6030	4	37
BN418_0007|8065	5	34
BN418_0008|10728	4	36
BN418_0009|12090	5	39
BN418_0010|12750	4	42
BN418_0011|13675	4	40
BN418_0012|14636	4	35
BN418_0013|16051	5	37
BN418_0015|17154	5	35
BN418_0016|19121	6	38
BN418_0017|19734	5	35
BN418_0019|C21231	5	38
BN418_0020|21457	4	36

*First column is some of sequences name; second column is the number of matched sites (Nm) between the SD sequence and the 50mer, and the last column is the start of the match (Sm).

**Table 7:** Statistical analysis of the SD sequences in *Listeria monocytogenes* La111 and *Klebsiella pneumoniae* KCTC 2242

***Klebsiella pneumoniae*** ** KCTC 2242**	***Listeria monocytogenes*** **La111**	**Statistical analysis**
4444	2860	N_gene_
3830	2600	N_SD_
0.8618	0.9090	P_SD_
4.4862	4.5846	Average of N_m_
0.5736	0.5897	Standard deviation of N_m_

*First column is some of sequences name; second column is the number of matched sites (Nm) between the SD sequence and the 50mer, and the last column is the start of the match (Sm).

## Conflict of interest

The author declared no conﬂicts of interest.

## References

[1] Beebe JL, Koneman EW (1995). Recovery of uncommon bacteria from blood: association with neoplastic disease. Clin Microbiol Rev.

[2] Schlech WF (2000). Foodborne listeriosis. Clin Infect Dis.

[3] Goulet V, Marchetti P (1996). Listeriosis in 225 non-pregnant patients in 1992: clinical aspects and outcome in relation to predisposing conditions. Scand J Infect Dis.

[4] Sauders BD, Wiedmann M, Desjardins M (2001). Recurrent Listeria monocytogenes infection: relapse or reinfection with a unique strain confirmed by molecular subtyping. Clin Infect Dis.

[5] Lin YT, Liu CJ, Fung CP ( 2011). Nosocomial Klebsiella pneumoniae bacteraemia in adult cancer patients--characteristics of neutropenic and non-neutropenic patients. Scand J Infect Dis.

[6] Henao-Martinez AF, Gonzalez-Fontal GR, Castillo-Mancilla JR (2013). Enterobacteriaceae bacteremias among cancer patients: an observational cohort study. Int J Infect Dis.

[7] Wurtzel O, Sesto N, Mellin JR ( 2012). Comparative transcriptomics of pathogenic and non-pathogenic Listeria species. Mol Syst Biol.

[8] Uemura S, Dorywalska M, Lee TH ( 2007). Peptide bond formation destabilizes Shine-Dalgarno interaction on the ribosome. Nature.

[9] Shine J, Dalgarno L ( 1974). The 3'-terminal sequence of Escherichia coli 16S ribosomal RNA: complementarity to nonsense triplets and ribosome binding sites. Proc Natl Acad Sci U S A.

[10] Steitz JA, Jakes K (1975). How ribosomes select initiator regions in mRNA: base pair formation between the 3' terminus of 16S rRNA and the mRNA during initiation of protein synthesis in Escherichia coli. Proc Natl Acad Sci U S A.

[11] Hui A, de Boer HA (1987). Specialized ribosome system: preferential translation of a single mRNA species by a subpopulation of mutated ribosomes in Escherichia coli. Proc Natl Acad Sci U S A.

[12] Jacob WF, Santer M, Dahlberg AE ( 1987). A single base change in the Shine-Dalgarno region of 16S rRNA of Escherichia coli affects translation of many proteins. Proc Natl Acad Sci U S A.

[13] Li GW, Oh E, Weissman JS (2012). The anti-Shine-Dalgarno sequence drives translational pausing and codon choice in bacteria. Nature.

[14] Motalleb G ( 2013). Translation Elongation Rate Measurement of Epstein-Barr virus Strain GD1. Iran J Cancer Prev.

[15] Xia X ( 2012). Position weight matrix, gibbs sampler, and the associated significance tests in motif characterization and prediction. Scientifica (Cairo).

[16] Jiong Ma, Campbell A, Karlin S ( 2002). Correlations between Shine-Dalgarno Sequences and Gene Features Such as Predicted Expression Levels and Operon Structures. J Bacteriol.

[17] Gold L ( 1988). Posttranscriptional regulatory mechanisms in Escherichia coli. Annu Rev Biochem.

[18] Xia X (2000). Data Analysis in Molecular Biology and Evolution.

